# Supplementary material for: Design and Clinical Application of an Integrated Microfluidic Device for Circulating Tumor Cells Isolation and Single-Cell Analysis
Source: Micromachines (Basel). 2021 Jan 2;12(1):49. doi: 10.3390/mi12010049 (PMC7824094; doi:10.3390/mi12010049)
Supplement: Supplementary file 1 [file micromachines-12-00049-s001.pdf]

micromachines

Supplementary Materials: Design and Clinical Application of an Integrated Microfluidic Device for Circulating Tumor Cells Isolation and Single-Cell Analysis

Mingxin Xu, Wenwen Liu, Kun Zou, Song Wei, Xinri Zhang, Encheng Li and Qi Wang

Table S1. Capture rate of the integrated device under different concentrations of PC-9 cells.

PC-9 cell concentrations (cells/mL)	Capture rate (%) of 3 independent tests			Average capture rate (%)
	1	2	3	
10^1	78.6	83.3	90.0	84.0
10^2	83.0	85.6	86.0	84.8
10^3	85.0	85.1	86.0	85.4
10^4	83.2	87.9	86.5	85.9

Legend to Table S1. 10^1–10^4 indicates the number of PC-9 cells spiked into healthy blood samples.

Table S2. Capture purity of the integrated device at a concentration of 104 cells/mL of PC-9 cells.

PC-9 cell concentrations (cells/mL)	Capture purity (%) of 3 independent tests			Average capture purity (%)
	1	2	3	
10^1	0.1	0.2	0.1	0.2
10^2	1.3	1.9	1.1	1.4
10^3	12.4	16.4	9.8	12.9
10^4	58.5	68.3	54.5	60.4

Legend to Table S2. 10^1–10^4 indicates the number of PC-9 cells spiked into healthy blood samples.

Table S3. Cell loss rate of the backwashing step under different concentrations of PC-9 cells.

PC-9 cell concentrations (cells/mL)	Cell loss rate (%) of 3 independent tests			Average cell loss rate (%)
	1	2	3	
10^1	14.3	11.1	10.0	11.8
10^2	10.4	12.8	9.0	10.7
10^3	10.4	9.6	9.2	9.7
10^4	6.2	7.5	8.1	7.3

Legend to Table S3. 10^1–10^4 indicates the number of PC-9 cells spiked into healthy blood samples.
